# Circulating Blood Lymphocytes and Acute Pancreatitis Severity: A Systematic Review

**DOI:** 10.7759/cureus.47532

**Published:** 2023-10-23

**Authors:** Filipa Malheiro, Margarida L Nascimento, Ana Carmo, Luis Miguel Borrego

**Affiliations:** 1 Internal Medicine, Hospital da Luz Lisboa, Lisboa, PRT; 2 Internal Medicine, Hospital Da Luz Lisboa, Lisboa, PRT; 3 Immunology, Hospital da Luz Lisboa, Lisboa, PRT; 4 Immunology, Nova Medical School, Lisboa, PRT

**Keywords:** systematic review, t cell, b cell, peripheral blood, lymphocytes, severity, acute pancreatitis

## Abstract

Acute pancreatitis is an acute inflammatory process of the pancreas with high prevalence and varying degrees of severity that can be potentially life-threatening. Much is still unknown about which mechanisms determine the course and severity of acute pancreatitis. The primary objective of this review is to identify the potential association between circulating lymphocytes and the severity of acute pancreatitis. A systematic search was performed in Medline, Web of Science, Cochrane Central Register of Controlled Trials and ClinicalTrails.gov. The authors independently did the selection process as well as data extraction that was recorded into a flow diagram following the Preferred Reporting Items for Systematic Review and Meta-Analysis Protocols (PRISMA-P). Our initial search identified 27,783 studies which were narrowed down to 13 by applying strict inclusion and exclusion algorithms. The consistent findings across the studies indicated that peripheral blood lymphocytes are related to acute pancreatitis severity.

## Introduction and background

Acute pancreatitis (AP) is an inflammatory disease of the pancreas with an unpredictable course. This is one of the reasons why it is a leading cause of hospitalization from gastrointestinal diseases in Europe and the United States with significant morbidity, pancreatic insufficiency, and long-term illness [1,2]. We now know injured acinar cells of the pancreas release chemokines and cytokines leading to infiltration of immune cells and worsening tissue injury of the pancreas and later systemic inflammation [3]. Acute pancreatitis is characterized by local and systemic inflammation and the severity of this disease is associated with the systemic inflammatory response syndrome (SIRS) [4]. The activation of the innate immune system has been well described in acute pancreatitis, involving multiple mediators that lead to a cascade of inflammation and the subsequent activation of the adaptative immune system [5]. Several studies have tried to relate the severity of acute pancreatitis and the different components of the innate immune system including c-reactive protein and cytokines [6]. Other studies have tried to relate the severity of acute pancreatitis and the different components of the adaptative immune system including cytokines and lymphocytes [7,8]. B and T cells possibly play critical roles in the pathogenesis and severity of acute pancreatitis although their exact role has not yet been elucidated [9,10]. To date, to our knowledge, there are no systematic reviews relating peripheral blood lymphocytes and the severity of acute pancreatitis. Therefore, this systematic review will focus on the role of these cells in the severity of acute pancreatitis.

## Review

Methods

The Preferred Reporting Items for Systematic Review and Meta-Analysis Protocols (PRISMA-P) was followed in this systematic review [11]. The study protocol has been pre-registered on PROSPERO (registration number CRD42023383303). The main objective of this work is to systematically review and summarize the current knowledge on the potential association between circulating lymphocytes and relate it to the severity of acute pancreatitis.

Eligibility Criteria

This study identified cohort studies and case-control studies that relate blood lymphocytes with the severity of acute pancreatitis. Cross-sectional studies, case series and case reports were excluded. We included only articles reported in the English language. Only studies on the adult human population (18 years and older) were included. No restriction regarding publication was set. Therefore, studies were included from inception to 31st January 2022.

Intervention Exposure

Inclusion criteria: Human adults, hospitalized with the diagnosis of acute pancreatitis with blood collection to determine lymphocyte levels, percentage and/or absolute number. The diagnosis of acute pancreatitis required the presence of two of the following three criteria: acute onset of persistent, severe epigastric pain often radiating to the back; elevation in serum lipase or amylase three times or greater than the upper limit of normal; and characteristic findings of acute pancreatitis on imaging (contrast-enhanced computed tomography, magnetic resonance imaging, or transabdominal ultrasonography). The severity of acute pancreatitis was defined according to the classification of severity applied by The Atlanta Classification System 1992, The Revised Atlanta Classification System 2012 and/or the following scoring systems: the Acute Physiology and Chronic Health Examination (APACHE) II score, the Bedside Index of Severity in Acute Pancreatitis (BISAP) score, Ranson's criteria, and the Computed Tomography (CT) severity index. Exclusion criteria were less than 18 years old, non-human, non-hospitalized.

**Table 1:** PICOST criteria for the inclusion of studies into the systematic review PICOST: population, intervention, comparator, outcome, setting, timing

Criteria	Description
Participants	Adult human population (>18 years old)
Exposure	Hospitalized patients with acute pancreatitis
Comparator	Not applicable
Primary outcome	Relate peripheral blood lymphocytes and the severity of acute pancreatitis
Study Design	Cohort studies and case-control studies
Timing	From inception to 31^st^ January 2022

Search Strategy

A comprehensive computerized literature research strategy was conducted to find the studies to be included in this systematic review. Published and unpublished studies were searched from the following databases: PubMed/Medline, Web of Science and Cochrane Central Register of Controlled Trials. The electronic database search was supplemented by searching ClinicalTrials.gov for ongoing or unpublished clinical trials. The search included the following key words and all their variants, according to each database and its special requirements: “acute pancreatitis”, “severity”,” lymphocyte”, “B cell”, “T cell”, “immune”. Boolean operators like ‘OR’ or ‘AND’ were also used. This information is exemplified in Table 2.

**Table 2:** Search strategy for PubMed MeSH: Medical Subject Heading

Query	Search
1	“pancreatitis” (MeSH terms) OR “pancreatitis” OR “acute pancreatitis” (MeSH terms) OR “acute pancreatitis”
2	“severity” (MeSH terms) OR “severity” OR “severe” (MeSH terms) OR “severe”
3	“lymphocyte” (MeSH terms) OR “lymphocyte” OR “immune” (MeSH terms) OR “immune” OR “immune cell” (MeSH terms) OR “immune cell”
4	“T cell” (MeSH terms) OR “T cell” OR “CD4” (MeSH terms) OR “CD4” OR “CD8” (MeSH terms) OR “CD8”
5	“B cell” (MeSH terms) OR “B cell”
6	1 AND 2 AND 3 AND 4 AND 5

Study Selection

Two reviewers independently and blind to each other conducted the selection process. Published studies were imported to the Mendeley citation software where duplicates were managed and discarded. All records identified in the search stage were screened by title/abstract and those not matching the criteria were discarded. The remaining studies were fully reviewed and included or excluded according to the inclusion and exclusion criteria. All variables were collected on a Microsoft Excel sheet (Redmond, WA, USA). Data extraction included features of the study including methodology, patient’s characteristics, severity score applied, times of measurement and outcomes. Discrepancies between the reviewers were identified and solved by consensus.

Risk of Bias Assessment and Data Synthesis

To minimize bias in the methodological quality of all studies included in this systematic review, the studies were categorized according to their quality by two independent reviewers (blind to each other). The Newcastle-Ottawa Scale (NOS) was used to assess quality. This is a reliable and valid tool for quality assessment of case-control and cohort studies to be used in a systematic review. It applies a “star system” on three perspectives of case-control and cohort studies: the selection of the study groups; the comparability of the groups; and the ascertainment of either the exposure or outcome of interest respectively. 

Results

**Figure 1:**
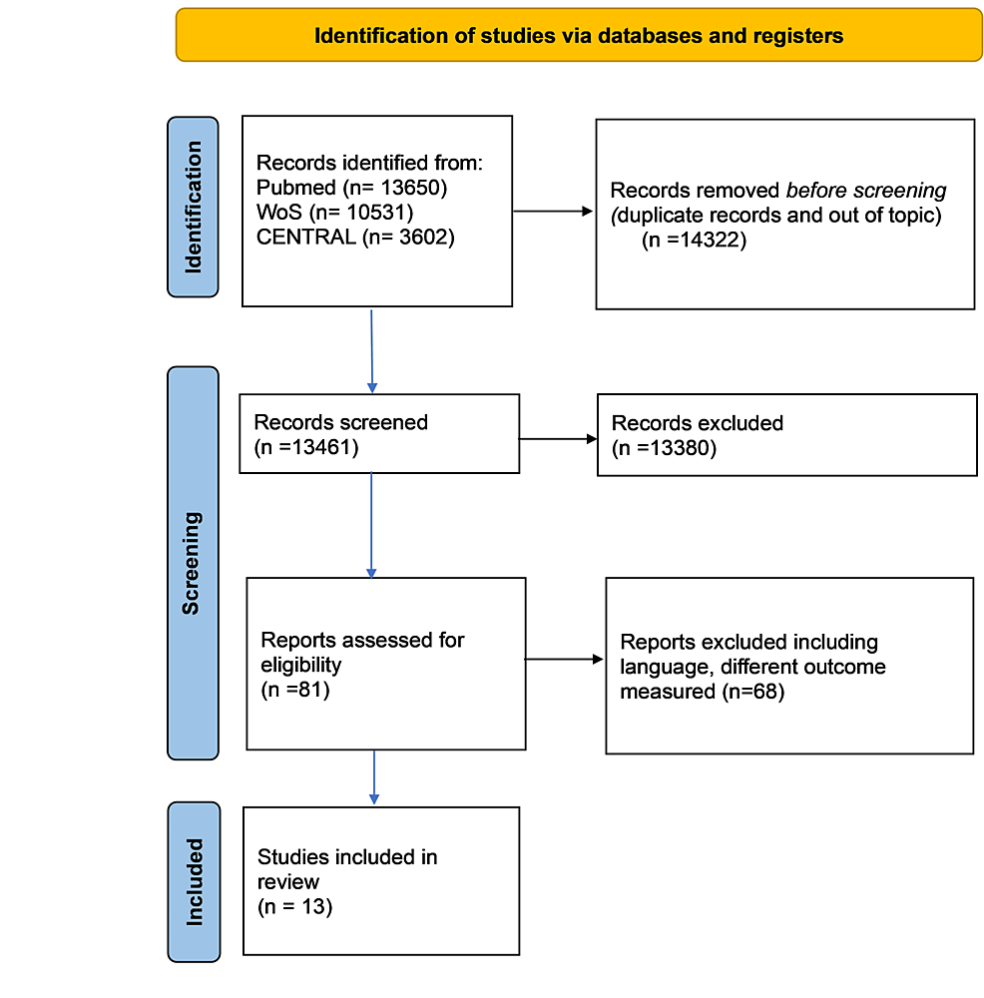
PRISMA flow diagram of screening process PRISMA: Preferred Reporting Items for Systematic Reviews and Meta-Analyses, WoS: Web of Science

In total, 27,783 studies were screened of which 13 studies were included in the final study. Of these 13 studies, five were case-control studies and eight were cohort studies. The selection process as well as data extraction are recorded into a flow diagram (Figure 1).

Some studies did not report sufficient details and of the 13 studies three had low quality assessment according to the NOS, five had fair quality assessment and five had good quality assessment (Table 2, 3).

**Table 3:** Newcastle-Ottawa quality assessment scale: case-control studies

Criteria	Qiu L et al. [9]	Widdison A et al. [12]	Wang Y et al. [13]	Pezzilli R et al. [14]	Galloway et al. [15]
Selection					
Case definition					
Representativeness of cases	*		*		
Selection of controls	*	*	*		*
Definition of controls			*		
Comparability	*		**	**	
Exposure					
Ascertainment of exposure					
Same method for cases and controls	*	*	*	*	*
Non-response rate	*	*	*	*	*
Total score of stars	5	3	7	4	3
Overall grade of quality	fair	poor	good	poor	poor

**Table 4:** Newcastle-Ottawa quality assessment scale: cohort studies

Criteria	Peng Y et al. [4]	Qi X et al. [16]	Liu X et al. [17]	Kan N et al. [18]	Jones M et al. [19]	Abayli B et al. [20]	Formanchuk T et al. [21]	Cao et al. [22]
Selection								
Representativeness of the exposed cohort	*	*	*	*	*	*	*	*
Selection of the non-exposed cohort	*	*	*	*	*	*	*	*
Ascertainment of exposure				*	*	*		
Demonstration outcome of interest was not present at start of study	*					*		
Comparability	**	**	**	**	**	**	**	**
Outcome								
Assessment of outcome					*			
Follow-up long enough for outcomes	*	*	*	*	*	*	*	*
Adequacy of follow up of cohorts	*	*	*	*	*	*	*	*
Total score of stars	7	6	6	7	8	8	6	6
Overall grade of quality	good	fair	fair	good	good	good	fair	fair

Data from eligible studies are presented (Table 4). One study applied the APACHE II score to divide patients in groups according to severity, one applied the CT severity index score, one applied the Ranson score and one the Atlanta 1992. All the other studies (total nine) applied the 2012 Revised Atlanta Classification. Most studies have considered the absolute number of lymphocytes in peripheral blood while others have presented the percentage and/or the absolute number of lymphocytes and the results have been similar.

**Table 5:** Characteristics of papers included in the review AP: acute pancreatitis, MAP: mild acute pancreatitis, MSAP: moderately severe acute pancreatitis, SAP: severe acute pancreatitis, HC: healthy controls, LR: lymphocyte ratio, CT: computed tomography, Bregs: B regulatory cells

Authors	Year of publication	Study design	Nº patients	Nº controls	Description and conclusions
Peng Y et al. [4]	2021	Cohort, retrospective	94	-	Patients were divided in two groups according to the Revised Atlanta Classification: mild and moderately severe AP (without persistent organ failure) and SAP (organ failure>48h). No difference was found in total blood lymphocytes between the two groups but B cells were positively related to the severity of disease and were a good predictor of persistent organ failure (SAP). T cells were negatively related to the severity of acute pancreatitis. The authors do not mention how and when the cytometry was made since this a retrospective study.
Qiu L et al. [9]	2018	Case-control, prospective	61	21	The number of lymphocytes was significantly lower in patients with acute pancreatitis on admission than those of HC but no significant difference was observed between patients with MAP and SAP according to The Revised Atlanta Classification System 2012. MSAP were not included in the study. Lymphocyte subsets were studied including Bregs and there were shown to predict the severity of AP.
Widdison A et al. [12]	1996	Case-control, prospective	20	20	Acute pancreatitis patients had significant lower values of lymphocytes in peripheral blood in the first 48h of admission than HC and SAP had significant lower values than MAP. The 1992 Atlanta Classification was used. T cells were also analyzed and were also significantly lower in patients than in healthy controls and significantly lower in SAP than in MAP.
Wang Y et al. [13]	2021	Case-control, prospective	103	62	The study showed patients with SAP according to The Revised Atlanta Classification System 2012 had significant lower values of blood lymphocytes on admission and MSAP also had significant lower values than MAP.
Pezzilli R et al. [14]	2003	Case-control, prospective	30	30	The Atlanta Classification System 1992 was used to classify patients according to severity and the control group included patients hospitalized with nonpancreatic acute abdominal pain. The percentage of lymphocytes was significantly lower in patients with acute pancreatitis than in controls and was also significantly lower in SAP than in the MAP group on admission.
Galloway et al. [15]	1994	Case-control, prospective	35	Not mentioned	Patients with AP were classified as severe and mild according to APACHE II. On admission AP patients had significant lower lymphocytes than healthy controls but no difference was found in lymphocyte count between patients with mild and severe AP.
Qi X et al. [16]	2017	Cohort, retrospective	200	-	Patients were classified as either SAP (including SAP and MSAP) or MAP using the Atlanta classification. On admission the lymphocyte count and the LR of the SAP cohort was significantly lower than the MAP group. The LR was found to predict the severity of AP.
Liu X et al. [17]	2021	Cohort, retrospective	101	-	Patients were divided into two groups MAP and SAP (includes MSAP and Severe Acute Pancreatitis of the Atlanta Classification). Patients in the SAP had significantly lower lymphocytes than patients in the MAP group.
Kan N et al. [18]	2021	Cohort, retrospective	154	-	Two study groups were identified based on radiological grading of severity of pancreatitis using the CT severity index score. Lymphocyte count on day one of hospitalization was negatively correlated with severity of acute pancreatitis.
Jones M et al. [19]	2017	Cohort, retrospective	629	-	Patients were classified in two groups according to The Revised Atlanta Classification: mild/moderate and severe acute pancreatitis. It was found the lymphocyte count was significantly lower in patients with severe acute pancreatitis on day two and three of hospitalization.
Abayli B et al. [20]	2018	Cohort, retrospective	435	-	Patients were classified in two groups of severity according to the Ranson score and the lymphocyte count on admission was significantly lower in patients with the highest score.
Formanchuk T et al. [21]	2022	Cohort, retrospective	229	-	All patients were divided into two groups depending on the degrees of severity: the group with mild acute pancreatitis and another group which combined moderately severe AP and severe AP. A significant difference on lymphocyte percentage was found between the groups on admission (lower on the most severe group) between the three groups.
Cao et al. [22]	2021	Cohort, retrospective	571	-	According to The Revised Atlanta Classification the included subjects were classified into severe AP and non-severe AP (including patients with mild AP and patients with moderately severe AP). The percentage of lymphocytes (first 48hours of hospitalization) was significantly lower on the severe group of patients. A normogram using six indicators was established to predict AP severity which includes the percentage of lymphocytes.

Two case-control studies did not show a significant relation between blood lymphocytes and acute pancreatitis severity on admission but they both show significant lower lymphocytes than controls. From the 10 cohort studies one cohort study did not find a significant difference between blood lymphocytes in the most severe group of patients with acute pancreatitis and the least severe group of patients with acute pancreatitis but the intermediate group of severity (the moderately severe group) was not included in the study. In conclusion, from the 13 studies 10 found that lower levels in peripheral blood of lymphocytes were related to the severity of acute pancreatitis.

Discussion

Acute pancreatitis is an inflammatory disease of the pancreas that might have an uncertain course and that can be fatal. Much is still unknown about which mechanisms determine its course and severity. This is why several risk scores as well as individual biomarkers and radiological scoring systems have been developed to predict outcomes. The Revised Atlanta Classification System, from 2012, defining clinical diagnosis, CT manifestations, and the disease course of acute pancreatitis, is the most widely used in clinical practice [23]. This classification, evaluating additional local or systemic complications as well as the presence and duration of organ failure, divides acute pancreatitis into mild acute, moderately severe acute, and severe acute pancreatitis. However, this classification is only made when acute pancreatitis is already evolving and frequently when some of its complications are well established [24]. A few other scoring systems including clinical and laboratory criteria have also been devised like the BISAP score and Ranson’s criteria [25]. Once more, most of these scoring systems require 24 hours to predict severity as several parameters are not easily available on admission. Therefore, early prediction of acute severity is still needed. It is also known that the innate immune system and the adaptative immune system play an important role in acute inflammatory diseases, namely sepsis. Lymphopenia and immunosuppression have already been described in patients with septic shock [26]. This is also true for other inflammatory diseases such as acute pancreatitis and a better understanding of the role of the cells of the adaptative immune system in acute pancreatitis might have a huge impact on the outcomes and future treatments of patients with acute pancreatitis.The neutrophil-to-lymphocyte ratio has been shown to be superior to white blood cell count in predicting adverse outcomes of acute pancreatitis [27]. Another study also showed that the neutrophil-to-lymphocyte ratio was related to acute pancreatitis severity [18]. The neutrophil-to-lymphocyte ratio has been shown to be a good indicator of severe disease in inflammatory diseases including sepsis and acute pericarditis. The main finding of this study was that patients with the severe form of acute pancreatitis have lower blood levels of lymphocytes during hospitalization than patients with mild acute pancreatitis. In this review we found only three studies that showed no relation between blood lymphocytes and the severity of acute pancreatitis although in the case-control studies (two) that did not find a significant difference between the most severe cases and the less severe cases of acute pancreatitis there were significant lower blood lymphocytes between acute pancreatitis patients and HC. The study by Peng et al. which is a cohort study that did not find any difference in blood lymphocytes between the most severe cases and the mild and moderately severe cases of acute pancreatitis even though blood T cells were negatively associated with AP severity and blood B cells were positively related with severity of disease. The authors mention data was collected retrospectively but do not explain how this was done when studying B cells and T cells as this requires analyzing cells in a short period of time after blood collection. The other study was the case-control study by Qiu et al. that showed no difference between patients with mild AP (MAP) and severe AP (SAP), but moderately severe acute pancreatitis patients were not included in the study. The other study was also a case-control study by Galloway et al. that did not show a difference between blood lymphocytes in the most severe cases and the mild ones even though there was a significant difference between healthy controls and patients with acute pancreatitis. This study describes little information about the control group, including their total number, how and where they were recruited, whether from community or hospitalized patients. Risk of bias assessment demonstrates fair or good quality for most included studies. This review has some limitations and because of heterogeneity a meta-analysis was not performed. There were variations in study populations, definitions of different outcomes, severity scores applied and blood sampling time points. Another point is the lack of report by some studies on the duration of symptoms before hospitalization. Only patients within the 48-72 hours of onset of symptoms should be included but most studies do not mention when symptoms started. Peripheral blood lymphocyte depletion in acute pancreatitis may result from both excessive apoptosis and migration to the site of inflammation [28]. Thinking about future directions in the prediction of acute pancreatitis, some studies included in this review already have focused on more specific subpopulations of lymphocytes. Other reports have also study more specific subpopulations of lymphocytes, mainly T regulatory cells and B regulatory cells which are known to downregulate inflammation [7,29,30].

## Conclusions

Acute pancreatitis is a severe and burdensome disease whose pathophysiology is not yet fully understood and whose course is still unpredictable at hospitalization. Therefore, accurate and easy-to-use predictors are essential and much-needed to define at-risk patients. This review is the first, to the best of our knowledge, to describe the association between peripheral blood lymphocytes and the severity of acute pancreatitis. This study analyses, after thorough screening, data from five case-control studies and eight cohort studies and we can conclude that lower peripheral blood lymphocytes during hospitalization in acute pancreatitis is related with disease severity. The study of lymphocyte's subpopulations might have a role in determining the severity of acute pancreatitis considering that in an ideal world we should have a single and specific biomarker that could predict with high accuracy the severity of acute pancreatitis.
